# Experiences of Siblings of Individuals with Autism Spectrum Disorders

**DOI:** 10.1155/2012/949586

**Published:** 2012-04-10

**Authors:** Maureen E. Angell, Hedda Meadan, Julia B. Stoner

**Affiliations:** Department of Special Education, Campus Box 5910, Illinois State University, Normal, IL 61790-5910, USA

## Abstract

The purpose of this study was to explore the experiences of siblings of individuals with autism spectrum disorders (ASDs) and identify their self-reported support needs. We conducted in-person semi-structured interviews with 12 siblings aged 7 to 15 of children aged 6 to 15 with ASDs. Employing a qualitative collective case study research method, we conducted cross-case analyses to address our research questions. Three major themes emerged: (a) descriptions of the sibling subsystem (b) cohesion between and among the siblings, and (c) adaptability of the participant siblings to having family members with ASDs. Discussion of these findings and recommendations for future research contributes to the existing literature on siblings of children with disabilities.

## 1. Introduction

 Autism spectrum disorders (ASDs) are a group of developmental disabilities characterized by atypical development in socialization, communication, and behavior [1]. The Centers for Disease Control and Prevention (CDC) reported that more children are being diagnosed with ASDs than ever before. The most recent data reported by the CDC show that about one in every 88 children has ASD [2]. Many individuals with ASDs have unique characteristics that could impact family members, including parents, siblings, and extended family members [3], and, therefore, researchers have been exploring the experiences of family members when children with ASDs are involved (e.g., [4]).

 Family systems theory has several basic assumptions: (a) family characteristics are inputs into the family system, to which the family responds, and from which outputs (i.e., family functions such as affection, daily care, etc.) are produced; (b) the family is a whole system and is affected by the relationships among family members, and (c) boundaries exist between family subsystems (i.e., parental, marital, sibling, and extended family) and with the outside world [5]. Family member interaction occurs within and across family subsystems, has varying levels of cohesion (emotional bonding) and adaptability (ability to change in demand to stressful situations), is a dynamic process, and provides children with their first interactions with others. Balanced levels of cohesion and adaptability are the most conducive to “healthy family functioning” [6, page 65]. For families with children with ASDs, the sibling subsystem is of heightened importance since the sibling relationship can be the first and most intense peer relationship [7].

Typical sibling relationships change over time and provide the siblings opportunities to experience sharing, companionship, rivalry, and other outcomes [8]. Some sibling pairs experience warm, supportive relationships, whereas others experience conflicts and isolation [9]. Some researchers have explored sibling relationships, adjustment, and outcomes when one sibling has an ASD (e.g., [10–16]). Meadan et al. [17] reviewed the literature related to the social, emotional, and behavioral adjustment of siblings of individuals with ASDs. The authors reported mixed results on outcomes for and adjustment of typically developing siblings of individuals with ASDs. Researchers investigating this topic have reported that some children are positively affected (e.g., have high levels of self-concept and social competence) by having siblings with ASDs or don't experience or report on negative effects, while other siblings experience negative effects (e.g., feelings of loneliness, low levels of prosocial behavior, and increased internalizing and externalizing problem behavior). The mixed and inconclusive results from the reviewed studies were suggested to be due, in part, to the varied methods, measures, tools, informants, and control-contrast groups used across the studies [10, 17, 18].

 Beyer [19] reviewed the literature related to siblings' relationships when one sibling has an ASD. Beyer reported that researchers found both positive and negative factors in these siblings' relationships. Siblings claimed that they had minimal conflicts and warm relationships with their siblings with ASDs [20]. They also stated that they had fun with their siblings with ASDs (e.g., [9]) and were proud of teaching their siblings with ASDs [21]. In contrast, some siblings of children with ASDs reported that their relationships with their brothers or sisters with ASDs were less close and warm compared to relationships with other siblings [22]. In addition, a few siblings reported greater feelings of embarrassment than siblings of typically developing children or children with other disabilities [16]. Similar to Meadan et al. [17], Beyer suggested that different research methods and comparison groups, in addition to variations in other factors (e.g., family environment or severity of the ASD) could explain the mixed results related to the siblings' relationships.

 Due to the potentially negative effects of having a sibling with an ASD, it is important to continue the research on this topic and to identify supports and strategies that could facilitate the development of positive relationships between the siblings with and without an ASD and promote positive outcomes and adjustment for typically developing siblings. Beyer [19] and Meadan et al. [17] also highlighted the need to further investigate the experience of siblings of children with ASDs, using different research methods (e.g., qualitative methodology) and exploring different factors (e.g., ages of siblings with and without ASDs). Mascha and Boucher [16] conducted a pilot study and interviewed 14 typically developing siblings of individuals with ASDs. The authors emphasized that their findings could “be considered preliminary only” (page 27). Petalas et al. [23] called for more reports of siblings' accounts of their experiences growing up in families with siblings with ASDs, filling the gap in the literature focused on the viewpoints and voices of siblings of children with ASDs in middle childhood and preadolescence.

The purpose of the current study was to investigate the experiences of siblings of individuals with ASDs and identify their self-reported support needs. The research questions that guided this inquiry were: (a) What is the experience and impact of having a sibling with an ASD from the perspective of a typically developing child? and (b) What type of support do siblings of individuals with ASDs perceive they need?

## 2. Method

### 2.1. Research Design

The research team consisted of three faculty members from the Department of Special Education in a Midwestern university. All faculty members have worked directly with individuals on the autism spectrum, conducted research on the relations of families with children with ASDs, and have been trained extensively in qualitative research methods. We believe strongly that we have a responsibility to provide a venue and a “voice” for our participants who entrust us with their perspectives. For this study we employed qualitative research methodology to explore siblings' perceptions and experiences. The framework for the study was a grounded theory approach, in which the researcher identifies categories and concepts that emerge from the data and links these concepts into theories [24]. Specifically, we conducted a collective case study as described by Stake [25] to address this study's research questions. Collective case study involves the study of more than one case in order to “investigate a phenomenon, population, or general condition” (page 437). Brantlinger et al. [26] contended that investigating multiple cases will lead to better comprehension and better theorizing. Miles and Huberman [27] also asserted that studying multiple cases gives the researcher reassurance that the events in only one case are not “wholly idiosyncratic” (page 172). Furthermore, studying multiple cases allowed us to examine siblings' perspectives across all cases and enabled us to better understand these perspectives through more powerful descriptions and explanations.

We used cross-case analysis as described by Miles and Huberman [27] to study each sibling (i.e., case) as a whole entity. All three researchers independently line-by-line coded each sibling's interview responses to increase the rigor of our coding. We then met as a group to conduct a comparative analysis of all 12 cases. We used a constant comparative method [28, 29] to compare cases and to refine, expand, or delete categories as needed. This type of coding procedure helped us stay in tune with the siblings' perspectives as we continually studied our interview data [28] and contributed to the rigor of our methods [26]. As we discussed any disagreements we had about emergent categories, we returned to the interview data to again ascertain the siblings' viewpoints, and continued this process until we agreed on all categories. This process of cross-checking our coding of the major categories provided “thoroughness for interrogating the data” [30, page 1116] and afforded us opportunities to enhance our insights into our coding.

### 2.2. Participants

 We recruited participants in this study by distributing information about the study to local support groups (i.e., parent support groups, a support group for adolescents with ASDs) and centers that provided services to children with ASDs. Parents contacted us directly if they were willing to allow their children to participate. The only eligibility requirement was to have a sibling with an ASD and all siblings in the home were invited to participate. Participants included 12 typically developing siblings (6 boys and 6 girls) of children with ASDs. For the purpose of this paper, we will refer to the typically developing siblings as *participant siblings* and to the siblings with ASDs as *siblings with ASDs*. Participant siblings' ages ranged from 7 to 15 years. The ages of the siblings with ASDs (10 boys and 1 girl) ranged from 6 to 15 years. Two of the participant siblings (RaeEllen and Ruby) were sisters who had a brother, Pete, with an ASD. Two siblings, Mitch and Cloe, were twins with brothers with ASDs. The siblings with ASDs were not considered study participants since they were not interviewed, but we have included collective descriptions of them since they were the focus of the interviews with their siblings. See Table 1 for a summary of participants' demographic characteristics.

We learned quite a bit about our participants during the 1 : 1 interviews. Our intent in this report is to summarize characteristics of our participant siblings, their families, their siblings with ASDs that became evident throughout the interviews. We don't assume generalization of our sample beyond these particular participant siblings.

### 2.3. Interviews

 A member of the research team conducted a semistructured, individual, face-to-face interview with each participant sibling at a place and a time convenient for the family. Parents and participant siblings signed parent permission and child assent forms prior to engaging in their interviews. Each interview lasted about 45 minutes and included the following open-ended questions: (a) Tell me about you and about your family; (b) Describe your relationships with_(name of brother/sister with ASD); (c) What is the best part of being a sibling to_(name of brother/sister with ASD)? (d) What are some difficult parts of being a sibling to_(name of brother/sister with ASD)? (e) What helps you handle the difficult parts/situations? and (f) Can you think of other things that may help you?

### 2.4. Data Analysis

 We audiotaped all interviews, transcribed them for analysis, and entered information such as time, date, and location. Each researcher independently line-by-line coded each interview, and we entered all codes into NVivo7 software [31]. We developed a coding system by noting words and phrases that represented topics and patterns in the data [32]. Next, we met as a team to discuss the codes, identify emergent themes, and reach concordance on the organization of major categories. We used a flexible standard of categories that is, we adjusted our categories as we analyzed in depth data from each case [33]. As categories emerged, we segmented the data into simpler, general categories in order to expand and tease out the data for interpretation [30, 33]. We used NVivo 7 [31], a data management software program, to organize the coded data and the themes that emerged from the data, and to retrieve the quotes that we used to illustrate participants' responses. Our documentation provides a clear audit trail that allowed us to “claim dependable and confirmable results” [26, page 201].

## 3. Findings

**Figure 1:**
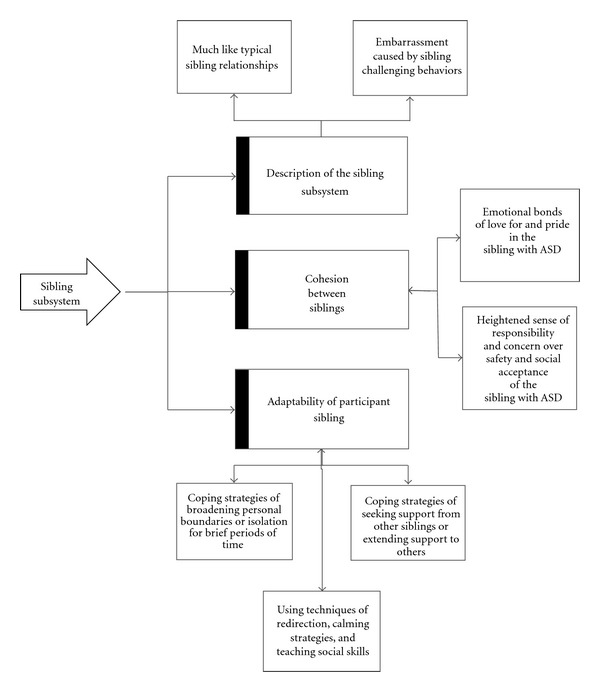
Graphic representation of the sibling subsystem.

 Our themes emerged from our data, and as we analyzed the relation among our major themes we found them to be reflective of family systems theory [5]. Specifically, our participant siblings described their sibling subsystem and sibling interactions which clearly reflected the tenets of cohesion and adaptability from family systems theory. Accordingly, we used the “lens” of family systems theory to analyze relations among our themes and the following three themes emerged: (a) descriptions of the sibling subsystem, (b) cohesion between and among the siblings, and (c) adaptability of the participant siblings to having family members with ASDs. These themes are interrelated, dynamic, and recursive in nature, as portrayed in family systems theory. For example, if a participant sibling described effective adaptability, s/he also described improvement in cohesion with a sibling with an ASD and/or described an improved relationship with that sibling. Consequently, we want to emphasize the connectedness among the themes we discuss and are providing a graphic representation of our findings in Figure 1.

### 3.1. Characteristics of Participant Siblings

Most of these participant siblings strove for academic success, wanting to do well in school. The older siblings were somewhat involved in their siblings' education—one had attended a parent/teacher conference; another volunteered at her sibling's summer camp. Socially, these participant siblings ranged from one who described herself as a “social butterfly” to those who said they had no friends. Our sample included interviewees who were socially competent but who expressed the desire to have more friends, especially friends who understand their siblings with ASDs. One participant, Mitch, identified a friend who had a brother with an ASD and described this friend as an important part of his support system.

Our participant siblings also described, throughout their conversations with us, the roles they played in their siblings' lives, the personal qualities they possessed, and the behaviors they engaged relative to their siblings with ASDs. Among the roles they reported were responsible caregivers, siblings' helpers, entertainers when their siblings needed to be redirected or occupied, “rescuers” when their siblings were aggressive, and parents' helpers.

Some of the personal qualities that emerged through our interviews were a caring nature, affection for siblings, acquiescence to siblings' demands, protection of siblings, feelings of responsibility for siblings' safety and well-being, compassion, patience, and persistence. One participant described herself as willing to get involved in her sibling's fixating interests. Another interviewee assumed responsibility for her sibling's miscommunication.

Our participant siblings recognized the talents and strengths of their siblings with ASDs as well as their needs. For example, one expressed his appreciation of his sibling's artistic talent; another, his sibling's musical talent. Although a few of the younger participant siblings demonstrated limited ability to explain autism, these siblings were generally aware of their siblings' disabilities and needs, although their understanding of their siblings' disabilities seemed to deepen with age. Some of them were also well aware of their parents' advocacy for their siblings. Specifically, they spoke about their siblings' diagnoses, social skill needs, medical and educational needs, needs for visual supports, and interventions in place to support their siblings. They also demonstrated awareness of their siblings' characteristics (physical and personality), personal preferences, and auditory or olfactory hypersensitivity. Even the youngest of our interviewees recognized their siblings' physical, intellectual, and behavioral differences from typically developing children. These siblings seemed to be aware of others' expectations for themselves and their siblings, especially in the area of social skills. One participant specifically expressed pride in her brother's improved social behavior.

### 3.2. Descriptions of the Sibling Subsystem

 Much like typical sibling relationships, our participant siblings described their sibling subsystems or relationships with their siblings with ASDs with varying degrees of contentment and discontent. Some of the positive comments from our participants focused on minimal sibling disagreements, engagement in mutual activities, and friendships between the siblings. Hanna, age 8, described the best part of the relationship with her sister: “The best part is that we don't fight that much and we don't usually get in fights, and she's just a really good sister.” Mera, age 15, had a similar response.

Well, it's really no different than a normal brother and sister relationship, except we've never—there's no disagreements. We never have disagreements, we never fight, there's really nothing, we can get along and there's no problem just me and him, it's always fun, we always laugh. When he plays video games and stuff I play with him sometimes and it's, I don't know, to me it's no different than a normal brother and sister relationship, because I've never had a little brother that does not have autism so I don't really know how that is, but with my older brothers it's just, it's the same.

Our participants also spoke of the activities they engaged in with their siblings with ASDs. These activities included playing video games, board games, swinging, and taking trips. Responding to the question, “What do you like about your brother?” 7-year-old Cloe described her thoughts about engaging in mutual activities: “Playing with him, going on trips with him, and doing things with him.” Cloe was also one of the participants who identified her brother as “her friend.”

However, our participants also made negative comments regarding their relationships with their siblings with ASDs and these centered on their siblings' challenging behaviors and the resulting embarrassment when inappropriate behavior occurred in public places. They identified challenging behaviors as the most negative aspect of having a sibling with an ASD. Our participants spoke of physical aggression, “meltdowns,” and verbal arguments. When describing his verbal arguments with his brother, Morris, age 7, stated, “We say stuff to each other that we kind of don't want to.” Cloe described the physical aggression her brother directed at her: “He squeezes me, bites me, and he pushes me.” Some participants described their siblings' physical aggression toward items they owned. For example, 10-year-old Mitch stated, “It's hard because he breaks my stuff a lot and I have to hide it, which is kind of not very fun.” The negative aspects were simply stated, freely shared, and often immediately followed by a positive comment, which appeared to be an attempt to reduce the negativity of their previous comments.

Another negative issue that arose was embarrassment when the siblings with ASDs did unusual things in public. Ruby, age 14, described an incident when this occurred:

There are times that my brother can say certain things in public that are supposed to be a secret between me and him. He will say them to other people or he will do things like that, that are annoying. He will run up to people that I like, certain teachers and be really loud in certain places, like the library. It will get the attention of everybody on you.

The resulting embarrassment was what seemed to bother the siblings the most when incidents occurred in public.

 Our participants compared their relationships with their siblings with ASDs to typical sibling relationships. They often spoke of incidents that were similar to those of typically developing siblings. For example, RaeEllen, age 12, described a practical joke she played on her brother:

Like one time he slept in until Noon, and it was Saturday. I told him he was late for school and his bus just left. He was like WHAT, OH NO! He got up and got in the shower and came back and got in the car. Then I went out and said, “Sorry, Pete, it is Saturday and lunch time.”

Frequently when describing their sibling subsystems the participant siblings clearly identified their relationships with their siblings as typical. For example, 11-year-old Breanna said, “So he is just a typical brother pretty much, annoying, gets in my business, and tries to talk to my friends.” Our participants described their sibling subsystems with positive comments, negative comments, and emphasis on the typical nature of their sibling relationships.

### 3.3. Cohesion between Siblings

 Cohesion in family systems theory refers to the emotional bonding between family members [5] and our participant siblings described cohesion with their siblings with ASDs as complex and varied. They expressed love, pride, a heightened sense of responsibility for their siblings with ASDs, and concerns for their siblings' social acceptance and safety. While a few participant siblings identified embarrassment over the behavior of their siblings with ASDs in public, many of them also identified pride in their siblings' accomplishments. For example, Ruby spoke of her embarrassment when her brother told others her secrets. Yet, she also was proud of having a relationship that allowed sharing secrets, “If Pete wasn't so unique I probably wouldn't have a brother I could talk to. My brother, I can tell secrets to and he can just be a very cool role model. It is amazing what he does.” Our participants spoke with pride of the many accomplishments of their siblings with ASDs. These accomplishments were varied and included memory skills, typing skills, and artistic ability. For example, 10-year-old Cabot spoke with pride of his brother's artistic ability, “He loves to draw. I think he's an awesome artist; although he does not think so.” The emotional bonding between our participants and their siblings with ASDs was also apparent in their responses. Breanna talked about her feelings when she was told that her brother had missed her, “My dad told me that night [when Breanna had attended a slumber party] he was asking about me. And I was like, “Oh, he's asking about me,” so I went and gave him a hug.” Ruby, age 14, described her brother as “one of the coolest people I know.”

 Perhaps due to their love of their siblings with ASDs, our participants also identified their sadness when their siblings and/or other children with disabilities were not socially accepted. RaeEllen described her feelings, saying, “I'm very sad at the way some kids are treated, with special needs.” Breanna, age 11, also spoke of her concern and empathy for her brother's lack of friends:

I don't know if it is worried or sad, but he does not have very many friends. He has never had a friend come over, or like most of his friends from school I know, because they are only 2 years younger than me, and I play with them sometimes. It makes me sad that he does not have more than a couple of friends.

Asia, age 14, expressed her concern about possible bullying at school:

I get nervous that kids would make fun of him in the halls. Whenever I heard that from kids in junior high, I always gave them a piece of my mind. I'm nervous that kids at school don't think before they speak. And they can really hurt feelings of kids like Nate who cannot understand them.

Another issue that dominated the feelings of our participants was the heightened sense of responsibility they felt for their siblings. When describing the care of his brother, Mitch, age 10, said, “Yeah. That's my job. Because it kind of falls on my shoulders a little bit. Um, it's a—just kind of like a responsibility.” Apparently, this responsibility weighed on Mitch enough that he addressed it with his mother:

I asked my mom. “I don't really want to do this all my life when I'm older; when I'm married, have kids.' And she said, “That's why we try to help and all that good stuff. And just, you're supposed to have your life and he's supposed to have his. We're not saying blow him off, but keep in touch, have help, be there once a week.” Yeah, okay.

The anxiety our participants had about their siblings' future also centered on the life skills their siblings with ASDs had not yet developed. Breanna had fears regarding her brother's safety due to his lack of communication skills:

Sometimes it is scary, because if someone would try to kidnap him, he wouldn't know what to do. He is not going to be able to scream and shout like me or somebody else would. It's kind of scary. Same with swimming. I get nervous if he is swimming by himself, like if me and my friends aren't out there or something. Like I do have occasional bad dreams about him drowning or dying because I cannot help him.

Fear, a heightened sense of responsibility, pride, and love dominated the feelings of our participants when they spoke of the cohesion with their siblings with ASDs.

### 3.4. Adaptability of Participant Siblings

 In family systems theory adaptability refers to the ability to change in terms of power structure, roles, and relationships in response to situational and developmental stress [5]. The adaptability of our participant siblings was clearly identified in response to the ASD characteristics of their siblings and resulted in personal coping strategies and techniques they used to ameliorate their siblings' challenging behaviors. We found that our participant siblings adapted in two distinct ways: (a) by using coping strategies and (b) by employing techniques that would assist their siblings with ASDs.

#### 3.4.1. Coping Strategies of Participant Siblings

We found that our participant siblings adapted to having siblings with ASDs by either isolating themselves, which reflected a restriction of their personal boundaries, or broadening their personal boundaries by obtaining support from others and/or actively educating others about ASDs. Specifically, restricting their personal boundaries through engagement in isolated activities gave our participants a break, a chance to remove themselves from either disturbing situations with their siblings with ASDs or just to be by themselves. A number of our participants spoke of retreating to their own rooms. Cloe, age 7, said “Basically my space is up in my room reading. I'm on beginner chapter books.” Morris, also age 7, talked about drawing to make himself feel better, “Well, one time I made this picture called candy world and that helped.” And James, age 8, said “I just usually go up to my room and watch TV for a while.” None of our participant siblings reported restricting their boundaries excessively—only when necessary and only for a brief period of time.

 Another coping strategy identified by our participants was obtaining support from others. Breanna, age 11, sought support from an online support group, and said:

Just talking about it, just talking about it makes me feel better. I don't know, it just makes me feel better that somebody else has someone that shares these same kinds of experiences. Not exactly the same, but similar kinds of experiences.

Asia, age 14, identified support from “talking with [her] friends” and 15-year-old Mera described support she received from another sibling of a child with an ASD, “I actually have a friend who has a cousin who is autistic, so I've talked to him about it. And my other friend's mom works here, actually, and I talk to her about it.” Mitch, age 10, also confided in a friend who had a brother with an ASD and felt solidarity from the shared experience:

I do have a friend; he used to be in my class. He still goes to my school. He has a brother with autism. I know his brother with autism. So, we were like, “Do you have a brother with autism? Yeah. I do too!” So we're like, “okay!” and if I wasn't, like, having a brother with autism, I would have been like, “What the heck are you talking about?”

Another coping strategy our participant siblings used was extending support to others. At one point in the interviews we asked about advice they would give to other siblings of children with ASDs. The most detailed response came from Asia, age 14, who said:

You just have to get used to it [autism] and don't think about all the negatives. Think about the positives. Think of all the advantages you have and not the disadvantages. Think of the bright side and think you are lucky they [siblings with ASD] can do as much as they can. Like some kids at the camp are in wheelchairs and are non-verbal, but then there are these kids with autism and you think, “I am so lucky.” With all the frustrations, you learn to get over it as you get older. So just deal with it now. You try not to get on your siblings' bad sides. As long as you are nice to them, they will be nice to you.

Our participant siblings identified support from others as a positive and valuable facet of their lives.

 Our participant siblings also reported actively participating in educating others about ASD. This occurred on both personal and broader-scaled levels. Jared, age 11, described helping his friend, who was a sibling of a child with an ASD, deal with his brother, “We'll, like, give each other ideas on what to do.” Mitch had written a book about his experiences of having a twin brother with an ASD and had spoken to several groups about his experience: “I get to share most stuff, like different stuff, about my brother. I get to tell a story about mine. Um, I get to give presentations about autism, um, in front of 2,518 people.” Ruby also found writing and speaking about her experiences helpful: “Actually I have written some things. I have been working with groups for a while now. In seventh grade, not only did I write a paper but that got published in a newsletter.”

#### 3.4.2. Techniques Used by Participant Siblings

Our participants spoke of adapting to the experience of living with siblings with ASDs by learning techniques that were effective when dealing with the challenges their siblings posed. They learned these strategies primarily from their parents and focused on techniques that calmed their siblings, redirected their siblings' attention, and/or taught their siblings new skills. Calming techniques usually occurred once a meltdown was in progress or immediately after. Hanna, age 8, described her calming technique with her sister, “So I don't try to get too close to her—I'm just, whenever I like, scratch her on her back she kind of calms down.” Other participants talked about using calm voices and modulated tones to help calm their siblings with ASDs.

 Our participant siblings frequently used redirecting techniques to redirect their siblings' attention. This redirecting usually occurred before potential meltdowns. Jared described his use of this technique:

Well, I actually go, like, probably in front of him, or something like that to like, try to get him to like, look at something else or something. I usually say, like, “Keep walking” or something like that. Or, like, “If you be good, you can probably rent a movie on the way back” and he'll go, “Oh!”

This appeared to be an effective use of the Premack principle which states that more probable or preferred behaviors will reinforce less probable or less preferred behaviors [34, 35]. Mitch used this technique, also: “Like, saying, “We just have to do this, but then we can get a Sprite, pretzel, cheese.””

 Teaching their siblings new skills was also a technique that our participants used in a proactive manner. It appeared that the siblings chose this strategy to provide their siblings with ASDs with skills that might prevent future challenging behaviors. RaeEllen spoke of trying to improve her brother's social skills:

I'm sort of working with him on how to be social. I'm a social butterfly. I'm a social person. We got some coffee there, at the Union, with an autistic boy. I'm trying to teach my brother how to set up a “play date.”

Cloe, age 7, was trying to teach her brother new vocabulary words which she qualified, “Like good things—not bad words or anything like that.” Hanna said that, in response to her sister's continuous repetition of phrases, “sometimes I say, “It kind of is funny, but don't say it all the time, like that might be kind of annoying.”” These participants were trying to teach their siblings skills that could be used to assist them socially or avert embarrassing social incidents.

## 4. Discussion

This study contributes to the qualitative research literature on the perspectives of typically developing siblings of children with ASDs on their experiences. More specifically, our findings, interpreted through the framework of family systems theory, offer insight into the sibling subsystem, cohesion between typically developing siblings and siblings with ASDs, and adaptability of typically developing siblings. Healthy family functioning has been identified as balanced cohesion and adaptability [5, 6], and our data analysis offered insight into the self-reported cohesion and adaptability of our participant siblings.

It is important to note that due to the limited number of participants and the nature of this study (i.e., qualitative methodology) generalization of the findings to other siblings of children with ASDs is limited. We have organized our discussion to address our research questions.

### 4.1. What is the Experience and Impact of Having a Sibling with an ASD from the Perspective of a Typically Developing Child?

Recent research and reviews of literature on siblings' perspectives and their relationships within families that include children with disabilities have indicated that various contextual factors such as knowledge about the children's disabilities or conditions (e.g., [36–38]) and families' coping styles and resources (e.g., [23, 39–41]) may influence the experience of living with a sibling with an ASD.

The present study confirms the findings of Petalas et al. [23] whose interviews with eight typically developing siblings in middle childhood who had brothers with ASDs yielded several themes of both positive and negative valence consistent with ours: a general acceptance of the siblings with ASDs (e.g., feelings of pride and appreciation for siblings with ASDs), identification (to varying extents) of positive aspects of having brothers or sisters with ASDs, social isolation, the need to change their behavior to cope with siblings' idiosyncratic behaviors, reduced recreational time with families, and derivation of social support from a variety of sources. In addition, our participant siblings also described their sibling subsystems in relation to their cohesion and adaptability to their siblings with ASDs.

Some researchers have found that sibling cohesion is negatively affected by the presence of children with disabilities in families [9, 20, 42], and some have recently reported positive sibling cohesion (e.g., [23]). Other researchers have published mixed reports on sibling cohesion (e.g., [43–45]). Our current data support mixed findings. On a positive note, our interviewees told us that that they infrequently quarreled with their siblings, that they enjoyed mutual activities, and that they were friends with their siblings. However, our findings also confirm some of the negative reports on sibling relationships that include children with disabilities. Negative comments of our sibling participants focused on their embarrassment or frustration with their siblings' aggressive or socially inappropriate behavior.

 Our younger participants (Cloe, age 7, Morris, age 7, Hannah, age 8, and James, age 8) who were below the age of 10 described both negative and positive feelings but it was our older participants who provided more complex descriptions of their feelings and indicated that they were increasingly concerned with social acceptance for their siblings with ASDs and their own sense of responsibility regarding safety and care. Our findings confirmed those of Benderix and Sivberg [46], Mascha and Boucher [16], and Ross and Cuskelly [47] whose participants described the negative effects of being exposed to their siblings' sometimes frightening aggressive behavior. We also found siblings expressing feelings of embarrassment, frustration and anger as did the siblings in Petalas et al.'s [23] study.

Our findings confirm those of Stalker and Connors [48] who found higher levels of empathy and patience in siblings of individuals with disabilities. Our sibling interviewees also expressed a positive outlook on their family conditions and the futures of their brothers and sisters with ASDs. These comments appeared in participant responses regardless of their age, and many of our participants offered positive comments about their siblings after they had described negative incidents or attributes.

Benderix and Sivberg [46] conducted interviews with 14 siblings (aged 10-11) of children with moderate to severe intellectual disabilities or autism about their family experiences.

Their study yielded seven thematic categories related to the siblings' feelings about their family experiences. Different contextual factors influenced the siblings in our current study yet our findings related to our participant siblings' feelings (e.g., pride in the accomplishments of their siblings with ASDs, a heightened sense of responsibility for their siblings, concerns about their siblings' social acceptance and safety, frustration, and embarrassment in some social situations) are similar to Benderix and Sivberg's findings. However, our participant siblings' brothers and sisters all had ASDs; our sample did not include siblings of children with intellectual disabilities.

Unlike Benderix and Sivberg's participants, our interviewees did not face the impending placement of their siblings into group homes; however, they did share similar feelings of unsafety and anxiety when their siblings manifested aggressive behavior. Our research also extends Benderix and Sivberg's research by yielding data on siblings' adaptability through the use of coping strategies and employing techniques with their brothers and sisters with ASDs.

### 4.2. What Type of Support Do Siblings of Individuals with ASDs Perceive They Need?

 The answer to this research question is relatively simple; our participants wanted time for themselves when situations were stressful, and they benefitted from talking with others who understood their situation, educating others about ASDs, and implementing techniques to assist their siblings with ASDs. This study extends the limited research (e.g., [23, 47, 49]) on the adaptability of children living with siblings who have ASDs by identifying coping strategies. Two types of coping strategies are described in the literature: problem-focused coping strategies and emotion-focused coping strategies. Using problem-focused strategies, a person attempts to solve a problem or change the situation, whereas when using emotion-focused strategies a person attempts to manage or regulate emotional states produced by the stressor [50]. Our participants described both emotion-focused and problem-focused coping strategies. The coping strategies that our younger participants, below the age of 10, identified were focused on removing themselves from their siblings to defuse potentially conflictual situations. As our participants aged, they described their coping strategies with more complexity. Their coping strategies often involved seeking social support which is one of the five coping strategies that McCubbin et al. [51] identified in their theoretical framework on resilience.

As with Petalas et al.'s [23] sample, our sibling participants adapted by eliciting support from various sources, for example, online support groups and chatting in person or by phone with friends. They appreciated the opportunity to share their feelings and thoughts with other siblings who experienced similar challenges and rewards in living with their brothers or sisters with ASDs. Bagenholm and Gillberg [43] found that some siblings could talk comfortably only with someone outside the home about their brothers or sisters with disabilities. None of our interviewees explicitly told us that they purposely sought support outside their homes, but we noted that they all described peers as their sources of support.

 In studying the adjustment of siblings of children with ASDs, Meyer et al. [39] found a positive correlation between the severity of ASDs and siblings' adjustment problems. Our participants' descriptions of their adjustment and coping strategies also reflected this relation, especially in light of maladaptive behaviors. Some of our interviewees said that they sought quiet time and space when they needed respite from their siblings. Consideration of siblings' explanations of the coping strategies they employ may be helpful in planning support group programs for these children.

Previous researchers have found, as we did, that typically developing siblings often help to manage the behavior of and teach their younger siblings with disabilities (e.g., [18, 47, 52, 53]), modeling appropriate social behaviors and functional skills for them (e.g., [54]) and engaging in prosocial interactions with them (e.g., [55, 56]). Our sibling participants told us that their parents had taught them some strategies to use to calm or redirect their siblings when needed and that they used positive reinforcement to encourage their siblings to exhibit prosocial behavior. They also directly taught their siblings new skills that helped them function more effectively in social situations.

## 5. Limitations and Scope of the Study

 Although we used stringent qualitative research methods for this study, we recognize that the validity of the findings may be affected by some limitations. The first limitation of this study is that we did not establish extended relationships with the participants. We interviewed each sibling only once. Multiple interviews would have been ideal; however, we feel that our interview data and our analysis of them provide a strong foundation for examination of these participants' perspectives on their experiences as siblings of children with ASDs. We also recognize that the generalizability of the findings may be limited by the nature of our participants. Although these findings are based on the perspectives of only 12 siblings from a small geographic area within one state, these participants ranged in age from 7 to 15 and reflected a variety of family lifestyles. We also did not include triangulation methods in this study as Guba and Lincoln [57] recommended for reducing researcher bias.

## 6. Recommendations for Research and Practice

We concur with Smith and Elder's [40] contention that more work needs to be done, especially in the area of intervention research to identify effective strategies that support typically developing children as they adapt to the challenges faced in living with siblings with ASDs. We recommend more exploration on strategies that facilitate cohesion among siblings, extending the work of researchers like Strain and Danko [58]. We also support Meyer et al.'s [39] call for more research on the assessment of the functioning of all family subsystems when children with ASDs are involved. Our findings only scratch the surface of the wealth of information practitioners, and researchers may learn from siblings of children with ASDs. We echo Petalas et al.'s [23] exhortation to apply these preliminary findings to larger-scale studies to shed light on siblings' experiences that may inform practice related to support services for these youth.

More research is also needed on the effects of various types and aspects of family systems [5] and the varied experiences of siblings within families of diverse cultures [38]. The perceptions our participant siblings shared with us confirm the findings related to the positive effects of growing up with siblings with disabilities, including high family cohesion and less sibling rivalry [20, 59, 60]. However, our children were only from Midwestern Caucasian families.

More longitudinal research on the effects of family members with ASDs on typically developing siblings is also necessary. Such longitudinal research would extend the research conducted by Orsmond et al. [8] on growing up with family members with ASDs and its effects on future caregiving relationships. Supportive programming for siblings of children with ASDs is necessary and valuable to families of children with ASDs.

## Figures and Tables

**Table 1:** Participants' demographic information.

Participant sibling name	Participant sibling age and gender	Sibling with ASD age and gender	Number of family members	Marital status of parents
Cloe	7, F (Twin)	7, M	3	Divorced
Morris	7, M	6, M	4	Married
Hanna	8, F	11, F	4	Married
James	8, M	7, M	10	Married
Cabot	10, M	12, M	5	Married
Mitch	10, M (Twin)	10, M	4	Divorced
Breanna	11, F	9, M	3	Divorced
Jared	11, M	14, M	4	Married
RaeEllen	12, F	15, M	5	Married
Ruby	14, F	15, M	7	Married
Asia	14, F	13, M	4	Married
Mera	15, M	9, M	6	Married

Note: M: male, F: female.
